# Supplementary material for: Joint Associations of Outdoor Nitrogen Dioxide and Temperature with Incident Adult-Onset Asthma in the United States
Source: medRxiv. 2026 May 21:2026.05.15.26353311. Preprint. [Version 1] doi: 10.64898/2026.05.15.26353311 (PMC13228754; doi:10.64898/2026.05.15.26353311)
Supplement: Supplementary file 1 [file NIHPP2026.05.15.26353311v1-supplement-1.pdf]

medRxiv preprint doi: https://doi.org/10.64898/2026.05.15.26353311; this version posted May 21, 2026. The copyright holder for this preprint (which was not certified by peer review) is the author/funder, who has granted medRxiv a license to display the preprint in perpetuity. This article is a US Government work. It is not subject to copyright under 17 USC 105 and is also made available for use under a CC0 license.

Supplementary Tables and Figures

Supplementary Table 1: Overall associations between NO_2 and adult-onset asthma risk in the All of Us Research Program[1, 2]

Nitrogen dioxide (NO_2) Concentrations	Cases	HR (95% CI)
Q1 (4.14 – ≤18.31 ppb)	2150	1.00 (Ref)
Q2 (>18.31 – ≤27.20 ppb)	2687	0.98 (0.92, 1.03)
Q3 (>27.20 – ≤31.21 ppb)	1936	0.85 (0.80, 0.91)
Q4 (>31.21 – 44.69 ppb)	2859	1.08 (1.02, 1.15)
Continuous (per 8 ppb)	9632	1.03 (1.01, 1.04)<br>P-trend=0.006

[1]Cox proportional hazards models were adjusted for age, sex, race, ethnicity, BMI, smoking status, smoking pack years, annual household income, educational attainment, annual O_3 levels, and annual $PM_{2.5}$ levels.

[2]Analyses involving NO_2 quartiles and continuous exposure models were restricted to participants with complete data.

Abbreviations: HR=Hazard Ratio; CI=Confidence Interval; Ref=Reference Group

**Supplementary Table 2: Overall associations between temperature and adult-onset asthma risk in the All of Us Research Program**[1, 2]		
**Exposure Category**	**Cases**	**HR (95% CI)**
Annual Maximum Temperature (per 1°C)	9632	1.01 (1.00, 1.02)<br>P-trend=0.035
Annual Average Temperature (per 1°C)	9632	1.01 (1.00, 1.01)<br>P-trend=0.074

[1]Cox proportional hazards models were adjusted for age, sex, race, ethnicity, BMI, smoking status, smoking pack years, annual household income, educational attainment, annual O_3 levels, and annual $PM_{2.5}$ levels

[2]Analyses involving quartiles and continuous exposure models were restricted to participants with complete data, resulting in a reduced analytic sample compared to the overall study population.

Abbreviations: HR=Hazard Ratio; CI=Confidence Interval

medRxiv preprint doi: https://doi.org/10.64898/2026.05.15.26353311; this version posted May 21, 2026. The copyright holder for this preprint (which was not certified by peer review) is the author/funder, who has granted medRxiv a license to display the preprint in perpetuity. This article is a US Government work. It is not subject to copyright under 17 USC 105 and is also made available for use under a CC0 license.

Supplementary Table 3: Associations of NO_2 and temperature with adult-onset asthma among various average annual household income groups in the All of Us Research Program[1, 2, 3]

	Low		Mid		Upper	
**Exposure Category**	**Cases**	**HR (95% CI)**	**Cases**	**HR (95% CI)**	**Cases**	**HR (95% CI)**
**Maximum Temperature**						
Low Levels of NO_2 and Low Levels of Annual Maximum Temperature	702	1.00 (Ref)	709	1.00 (Ref)	256	1.00 (Ref)
Low Levels of NO_2 and High Levels of Annual Maximum Temperature	809	1.29 (1.16, 1.44)	533	1.14 (1.00, 1.29)	167	1.04 (0.84, 1.29)
High Levels of NO_2 and Low Levels of Annual Maximum Temperature	359	1.31 (1.14, 1.49)	127	1.23 (1.01, 1.49)	58	1.28 (0.95, 1.72)
High Levels of NO_2 and High Levels of Annual Maximum Temperature	449	1.83 (1.61, 2.07)	177	1.16 (0.97, 1.38)	71	0.98 (0.75, 1.30)
**Average Temperature**						

medRxiv preprint doi: https://doi.org/10.64898/2026.05.15.26353311; this version posted May 21, 2026. The copyright holder for this preprint (which was not certified by peer review) is the author/funder, who has granted medRxiv a license to display the preprint in perpetuity.
This article is a US Government work. It is not subject to copyright under 17 USC 105 and is also made available for use under a CC0 license.

Low Levels of NO_2 and Low Levels of Annual Average Temperature	754	1.00 (Ref)	732	1.00 (Ref)	262	1.00 (Ref)
Low Levels of NO_2 and High Levels of Annual Average Temperature	757	1.28 (1.15, 1.43)	510	1.18 (1.05, 1.34)	161	1.11 (0.89, 1.37)
High Levels of NO_2 and Low Levels of Annual Average Temperature	204	0.99 (0.84, 1.16)	75	1.01 (0.79, 1.29)	38	1.17 (0.82, 1.65)
High Levels of NO_2 and High Levels of Annual Average Temperature	604	1.87 (1.68, 2.10)	229	1.27 (1.09, 1.49)	91	1.10 (0.85, 1.41)

[1]Cox proportional hazards models were adjusted for age, sex, race, ethnicity, BMI, smoking status, smoking pack years, educational attainment, annual O_3 levels, and annual $PM_{2.5}$ levels

[2]P-heterogeneity (maximum temperature and income): 3.9e-14; P-heterogeneity (average temperature and income): 5.4e-18

[3]Analyses stratified by income were restricted to participants with non-missing environmental, income, and covariate data; therefore, case counts differ from the overall analytic sample.

Income categories: low: below $75,000; mid: $75,000 to $150,000; upper: above $150,000

Interaction analyses:	RERI (95% CI)	AP (95% CI)	S (95% CI)
NO_2 x Max Temp - Low	0.23(-0.09,0.55)	0.13(-0.06,0.28)	1.38(0.88,2.29)

medRxiv preprint doi: https://doi.org/10.64898/2026.05.15.26353311; this version posted May 21, 2026. The copyright holder for this preprint (which was not certified by peer review) is the author/funder, who has granted medRxiv a license to display the preprint in perpetuity. This article is a US Government work. It is not subject to copyright under 17 USC 105 and is also made available for use under a CC0 license.

NO_2 x Max Temp - Mid	-0.21(-0.57,0.13)	-0.18(-0.55,0.10)	0.43(0.04,1.61)
NO_2 x Max Temp - Upper	-0.34(-0.90,0.16)	-0.35(-1.07,0.14)	Not defined
NO_2 x Avg Temp - Low	0.60(0.30,0.90)	0.32(0.17,0.45)	Not defined
NO_2 x Avg Temp - Mid	0.08(-0.29,0.42)	0.06(-0.25,0.30)	1.42(0.26,2.99)
NO_2 x Avg Temp - Upper	-0.18(-0.79,0.34)	-0.16(-0.81,0.27)	0.36(0.02,2.40)

Abbreviations: HR=Hazard Ratio; CI=Confidence Interval; Ref=Reference Group; RERI= Relative Excess Risk due to Interaction; AP= Attributable Proportion, S=Synergy Index.

Supplementary Table 4: Nitrogen dioxide, temperature, and adult-onset asthma risk in the All of Us Research Program:

medRxiv preprint doi: https://doi.org/10.64898/2026.05.15.26353311; this version posted May 21, 2026. The copyright holder for this preprint (which was not certified by peer review) is the author/funder, who has granted medRxiv a license to display the preprint in perpetuity.
This article is a US Government work. It is not subject to copyright under 17 USC 105 and is also made available for use under a CC0 license.

application of cell weighting to account for non-probabilistic sampling[1]

		Overall (Unweighted)	State, age, race, and Hispanic status	State, age, race, Hispanic status, education, and income
**Exposure Category**	**Cases**	**HR (95% CI)**	**HR (95% CI)**	**HR (95% CI)**
**Maximum Temperature**				
Low Levels of NO_2 and Low Levels of Annual Maximum Temperature	1918	1.00 (Ref)	1.00 (Ref)	1.00 (Ref)
Low Levels of NO_2 and High Levels of Annual Maximum Temperature	1928	1.13 (1.06, 1.21)	1.14 (1.02, 1.26)	1.21 (1.09, 1.36)
High Levels of NO_2 and Low Levels of Annual Maximum Temperature	729	1.28 (1.17, 1.40)	1.26 (1.11, 1.44)	1.29 (1.11, 1.50)
High Levels of NO_2 and High Levels of Annual Maximum Temperature	912	1.37 (1.26, 1.49)	1.26 (1.12, 1.41)	1.42 (1.25, 1.62)
**Average Temperature**				
Low Levels of NO_2 and Low Levels of Annual Average Temperature	2012	1.00 (Ref)	1.00 (Ref)	1.00 (Ref)
Low Levels of NO_2 and High Levels of Annual Average Temperature	1834	1.14 (1.07, 1.22)	1.15 (1.04, 1.28)	1.25 (1.12, 1.40)
High Levels of NO_2 and Low Levels of Annual	397	0.98 (0.88, 1.10)	1.00 (0.84, 1.19)	1.01 (0.84, 1.23)

medRxiv preprint doi: https://doi.org/10.64898/2026.05.15.26353311; this version posted May 21, 2026. The copyright holder for this preprint (which was not certified by peer review) is the author/funder, who has granted medRxiv a license to display the preprint in perpetuity. This article is a US Government work. It is not subject to copyright under 17 USC 105 and is also made available for use under a CC0 license.

Average Temperature				
High Levels of NO_2 and High Levels of Annual Average Temperature	1244	1.49 (1.38, 1.61)	1.35 (1.21, 1.50)	1.51 (1.34, 1.70)

[1]Cox proportional hazards models were adjusted for age, sex, race, ethnicity, BMI, smoking status, smoking pack years, annual household income, educational attainment, annual O_3 levels, and annual $PM_{2.5}$ levels

Abbreviations: HR=Hazard Ratio; CI=Confidence Interval; Ref=Reference Group

medRxiv preprint doi: https://doi.org/10.64898/2026.05.15.26353311; this version posted May 21, 2026. The copyright holder for this preprint (which was not certified by peer review) is the author/funder, who has granted medRxiv a license to display the preprint in perpetuity.
This article is a US Government work. It is not subject to copyright under 17 USC 105 and is also made available for use under a CC0 license.

Supplementary Table 5. Quantile-Adaptive Survival Screening (QaSIS) Results for Adult-onset Asthma Evaluating Nitrogen Dioxide (NO_2) and Maximum Temperature[1]		
**Rank**	**Variable**	**Exposure Category**
1	Smoking (Pack Years)	---
2	Annual ground-level ozone	---
3	Annual $PM_{2.5}$	---
4	Body mass index	---
5	Age	---
6	NO_2/Maximum Temperature	Low/High
7	NO_2/Maximum Temperature	High/Low
8	NO_2/Maximum Temperature	High/High
9	Sex	Female
10	Smoking Status	Ever Smoker
11	Racial Background	Black
12	Racial Background	Asian
13	Ethnicity	Not Hispanic or Latino
14	Annual household income	Mid
15	Annual household income	Upper
16	Highest educational attainment attained	Undergraduate
17	Highest educational attainment attained	College
18	Highest educational attainment attained	Advanced Degree

[1]QaSIS ranks covariates according to a quantile-based survival screening statistic derived from weighted quantile regression with inverse probability of censoring weights. Higher rank indicates greater marginal importance for early time-to-Adult-onset Asthma at $\tau = 0.98$. QaSIS is a screening method and does not estimate adjusted effect sizes or statistical significance.

medRxiv preprint doi: https://doi.org/10.64898/2026.05.15.26353311; this version posted May 21, 2026. The copyright holder for this preprint (which was not certified by peer review) is the author/funder, who has granted medRxiv a license to display the preprint in perpetuity. This article is a US Government work. It is not subject to copyright under 17 USC 105 and is also made available for use under a CC0 license.

**Supplementary Table 6. Quantile-Adaptive Survival Screening (QaSIS) Results for Adult-onset Asthma Evaluating NO_2 and Average Temperature[1]**		
**Rank**	**Variable**	**Exposure Category**
1	Smoking (Pack Years)	---
2	Annual ground-level ozone	---
3	Annual $PM_{2.5}$	---
4	Body mass index	---
5	Age	---
6	NO_2/Average Temperature	Low/High
7	NO_2/Average Temperature	High/Low
8	NO_2/Average Temperature	High/High
9	Sex	Female
10	Smoking Status	Ever Smoker
11	Racial Background	Black
12	Racial Background	Asian
13	Ethnicity	Not Hispanic or Latino
14	Annual household income	Mid
15	Annual household income	Upper
16	Highest educational attainment attained	Undergraduate
17	Highest educational attainment attained	College
18	Highest educational attainment attained	Advanced Degree

[1]QaSIS ranks covariates according to a quantile-based survival screening statistic derived from weighted quantile regression with inverse probability of censoring weights. Higher rank indicates greater marginal importance for early time-to-Adult-onset Asthma at $\tau = 0.98$.

QaSIS is a screening method and does not estimate adjusted effect sizes or statistical significance.

medRxiv preprint doi: https://doi.org/10.64898/2026.05.15.26353311; this version posted May 21, 2026. The copyright holder for this preprint (which was not certified by peer review) is the author/funder, who has granted medRxiv a license to display the preprint in perpetuity. This article is a US Government work. It is not subject to copyright under 17 USC 105 and is also made available for use under a CC0 license.

**Supplementary Table 7: Nitrogen dioxide (NO_2), maximum temperature, and adult-onset asthma risk in the All of Us Research Program: censored quantile regression method [1, 2]**	
**Exposure Category**	**βτ (95% CI)**
Low Levels of NO_2 and Low Levels of Annual Maximum Temperature	0.00 (Ref)
Low Levels of NO_2 and High Levels of Annual Maximum Temperature	-0.12 (-0.52, 0.83)
High Levels of NO_2 and Low Levels of Annual Maximum Temperature	-0.26 (-1.51, 0.40)
High Levels of NO_2 and High Levels of Annual Maximum Temperature	-0.22 (-1.20, 0.66)

[1]CQR models were adjusted for age, sex, race, ethnicity, BMI, smoking status, smoking pack years, annual household income, educational attainment, annual O_3 levels, and annual $PM_{2.5}$ levels

Abbreviations: CI=Confidence Interval; βτ=Beta Tau; Ref=Reference Group

[2]Maximum Temperature (tau = 0.02)

medRxiv preprint doi: https://doi.org/10.64898/2026.05.15.26353311; this version posted May 21, 2026. The copyright holder for this preprint (which was not certified by peer review) is the author/funder, who has granted medRxiv a license to display the preprint in perpetuity. This article is a US Government work. It is not subject to copyright under 17 USC 105 and is also made available for use under a CC0 license.

**Supplementary Table 8: NO2, average temperature, and adult-onset asthma risk in the All of Us Research Program utilizing a censored quantile regression method[1, 2]**	
**Exposure Category**	**βτ (95% CI)**
Low Levels of NO_2 and Low Levels of Annual Average Temperature	0.00 (Ref)
Low Levels of NO_2 and High Levels of Annual Average Temperature	-0.14 (-0.78, 0.55)
High Levels of NO_2 and Low Levels of Annual Average Temperature	0.03 (-1.70, 0.59)
High Levels of NO_2 and High Levels of Annual Average Temperature	-0.30 (-1.27, 0.36)

[1]CQR methods were adjusted for age, sex, race, ethnicity, BMI, smoking status, smoking pack years, annual household income, educational attainment, annual O_3 levels, and annual $PM_{2.5}$ levels.

Abbreviations: CI=Confidence Interval; βτ=Beta Tau; Ref=Reference Group

[2]Average Temperature (tau = 0.02)

medRxiv preprint doi: https://doi.org/10.64898/2026.05.15.26353311; this version posted May 21, 2026. The copyright holder for this preprint (which was not certified by peer review) is the author/funder, who has granted medRxiv a license to display the preprint in perpetuity. This article is a US Government work. It is not subject to copyright under 17 USC 105 and is also made available for use under a CC0 license.

Supplementary Figure 1: Distribution of A) annual smoking pack years and B) annual maximum temperature by state in the contiguous United States

A)

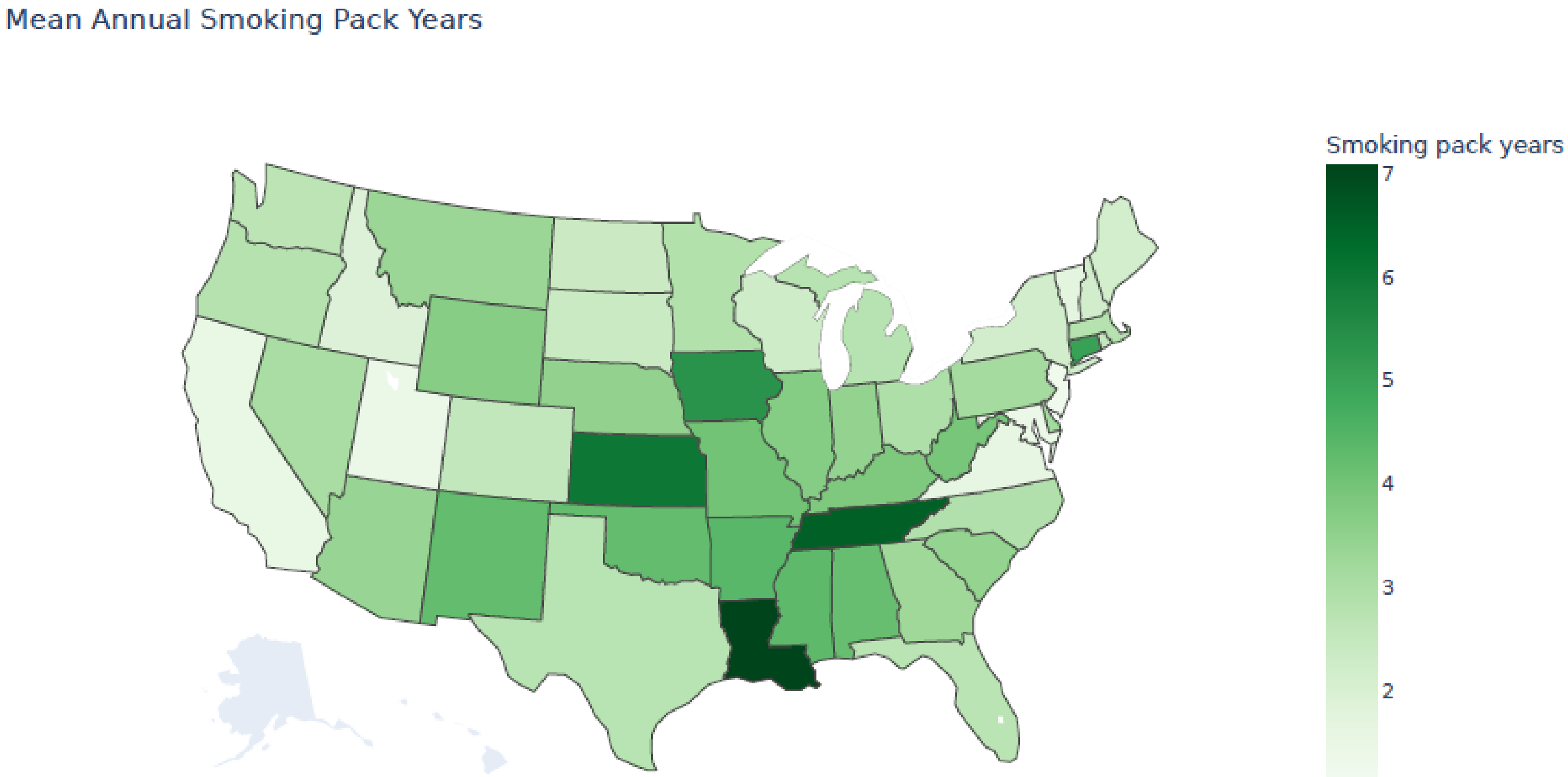

medRxiv preprint doi: https://doi.org/10.64898/2026.05.15.26353311; this version posted May 21, 2026. The copyright holder for this preprint (which was not certified by peer review) is the author/funder, who has granted medRxiv a license to display the preprint in perpetuity.
This article is a US Government work. It is not subject to copyright under 17 USC 105 and is also made available for use under a CC0 license.

B)

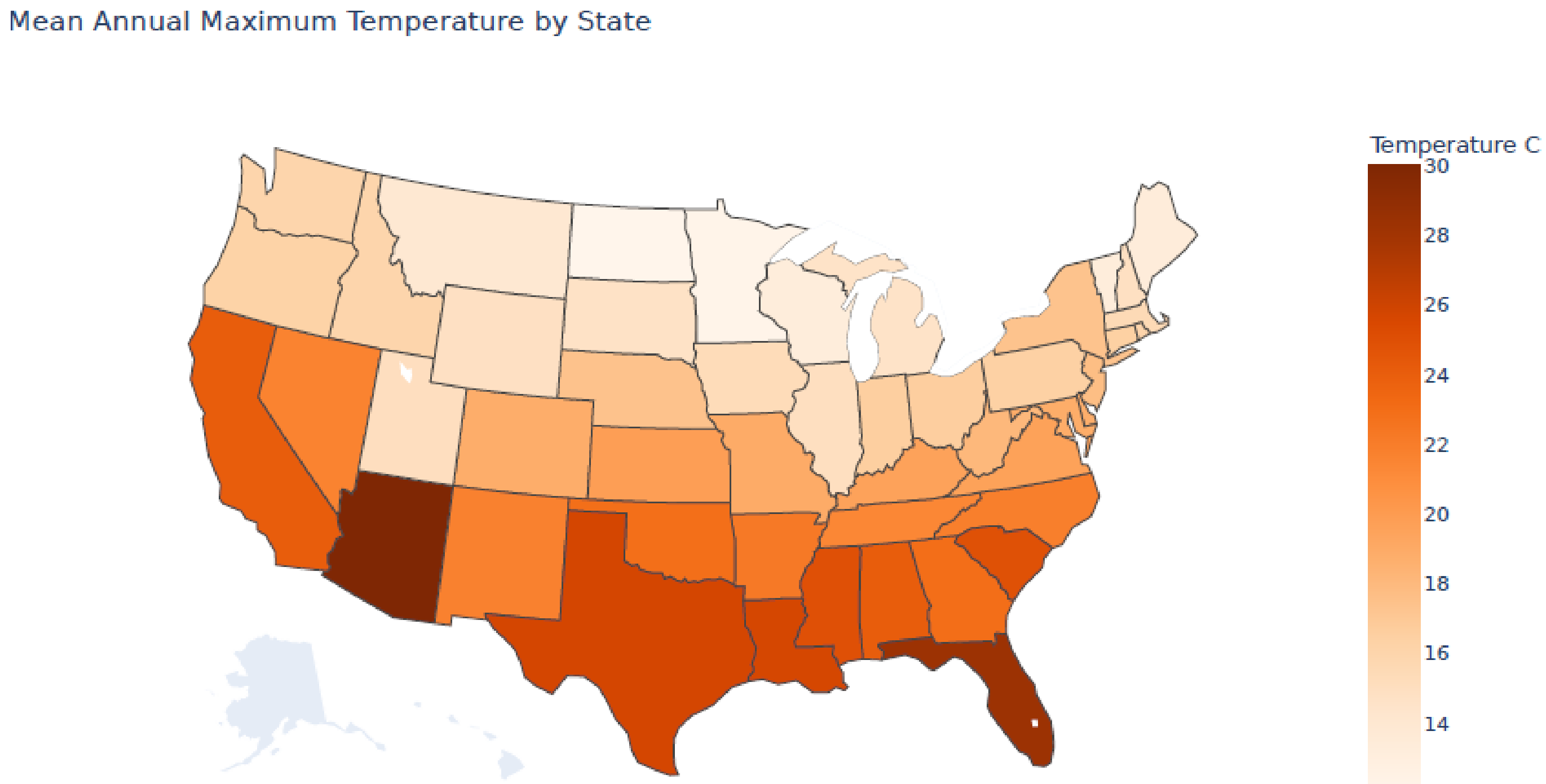

medRxiv preprint doi: https://doi.org/10.64898/2026.05.15.26353311; this version posted May 21, 2026. The copyright holder for this preprint (which was not certified by peer review) is the author/funder, who has granted medRxiv a license to display the preprint in perpetuity. This article is a US Government work. It is not subject to copyright under 17 USC 105 and is also made available for use under a CC0 license.
